# Correction to: ‘Identification of sex chromosome and sex-determining gene of southern catfish based on XX, XY and YY genome sequencing’ (2022) by Zheng *et al.*

**DOI:** 10.1098/rspb.2022.2381

**Published:** 2023-01-11

**Authors:** Shuqing Zheng, Wenjing Tao, Haowen Yang, Thomas D. Kocher, Zhijian Wang, Zuogang Peng, Li Jin, Deyong Pu, Yaoguang Zhang, Deshou Wang

*Proc. R. Soc. B* **289**, 20212645. (Published online 16 March 2022). (https://doi.org/10.1098/rspb.2021.2645)

In figure 3*c* of our recently published paper, ‘Identification of sex chromosome and sex-determining gene of southern catfish (*Silurus meridionalis*) based on XX, XY and YY genome sequencing’, the left part of image viii and the right part of image xvi show similar morphological structure. This may cause concerns about image duplication and alteration, and we wish to allay any concerns about the following explanation.

First, the two images referred to above are original, uncropped and otherwise unaltered (see the electronic supplementary material of the original paper). We have also included a flow chart (figure 1) to describe the imaging methods. As shown in the flow chart, successive sections of the same gonad were used to analyse the expression and cellular localization of *amhr2* and *amhr2y* in XX gonad at 120 dah by fluorescence *in situ* hybridization (FISH). The first section was hybridized with anti-sense probe of *amhr2y* and the second with anti-sense probe of *amhr2*. We used an Olympus FV3000 laser confocal microscope to take images with XY scan mode and 1 : 1 image size (aspect ratio). Therefore, the same area (circled by green dotted boxes) of the gonad would show similar, but not identical, morphological structure to neighbouring sections. We apologize for any confusion this may have caused.

**Figure 1.**
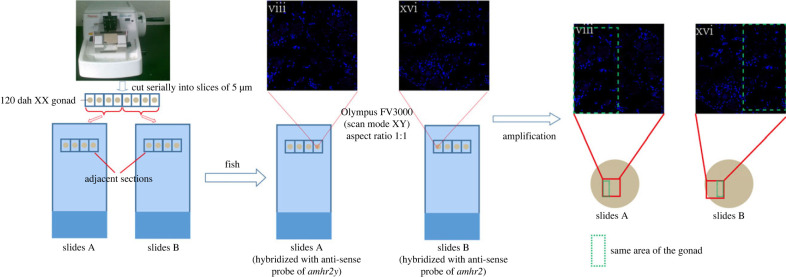
The flow chart showing the methods used for imaging. (Online version in colour.)

